# Detection of demodex mites in papulopustular rosacea using microscopic examination and polymerase chain reaction: a comparative case-control study

**DOI:** 10.1007/s00403-024-03228-1

**Published:** 2024-07-23

**Authors:** Ilaria Trave, Ilaria Salvi, Paola Canepa, Aurora Parodi, Emanuele Cozzani

**Affiliations:** Section of Dermatology, Department of Health Sciences, University of Genoa, IRCCS - Ospedale Policlinico San Martino, Genoa, Italy

**Keywords:** Rosacea, PCR, Demodex mites

## Abstract

Demodex mite proliferation is frequently involved in the pathogenesis of rosacea. The gold standard for Demodex identification is microscopic examination on a standardized skin surface biopsy. However, this method of sampling can be distressing and painful, especially when performed on hairy sites. In this case-control study, we compared the sensitivity of PCR and microscopic examination in diagnosing a Demodex infestation. Moreover, we investigated the possible correlations between the presence of Demodex mites and clinical characteristics. In total, 20 patients affected by papulopustular rosacea and 10 controls were included. At both microscopic examination and PCR, patients with rosacea presented a greater prevalence of positive samples than controls at the scalp and at the face. Microscopy had sensitivity of 50% at the face and of 46.7% at the scalp. PCR had sensitivity of 93.75% at the face and of 86.7% at the scalp. The positivity of PCR was associated to a higher frequency of facial papules and pustules. Patients with positivity at the face had a more frequent positivity at the scalp. The scalp could represent a reservoir for the Demodex mites, and should be investigated by sensitive and painless methods. PCR performed on painlessly collected samples should be further investigated.

## Introduction

Rosacea is an inflammatory skin disease with increase of erythema, papules and pustules which may be caused by Demodex mite proliferation [1–3]. For this reason, precise Demodex mite identification is necessary in order to improve the treatment of this inflammatory disease.

Traditionally, a standardized skin surface biopsy (SSSB) with microscopic examination is the gold standard for diagnosis [4]. During our last research, since we have demonstrated that the same Demodex mites (*Demodex folliculorum*) are increased not only at the face but also at the scalp of patients affected by papulopustular rosacea at the face, we reported the necessity to research for a painless sampling method to study Demodex mites at the scalp, where SSSB has showed to be painful and uncomfortable for patients [5].

PCR is a DNA-based strategy that can be used to identify pathogens by means of specific primers [6].

Our primary aim was to compare the sensitivity of PCR and microscopic examination in diagnosing a Demodex infestation on SSSB. In addition, we investigated the possible correlations between the presence of Demodex mites (diagnosed by PCR or microscopy) and clinical characteristics.

This is the first study comparing the sensitivity of PCR and microscopic examination in diagnosing a Demodex infestation. In addition, we investigated the possible correlations between the presence of Demodex mites (diagnosed by PCR or microscopy) and clinical characteristics.

## Materials and methods

In this case-control study, we included patients followed at the Dermatology Clinic (IRCCS, Ospedale Policlinico San Martino) affected by almost-clear, mild, and moderate papulopustular rosacea according to the current diagnostic criteria [7].

To assess disease severity, we applied the Investigator Global Assessment score (IGA score) [8]. Scalp signs (erythema, dandruff) and symptoms (itching, burning) of rosacea were evaluated in all patients. Patients with a history of, or affected by seborrheic dermatitis of the scalp, and patients with rosacea who had received antiparasitic treatments during the previous six months were excluded.

The patients were compared with healthy controls without a history of dermatologic conditions.

**Fig. 1:**
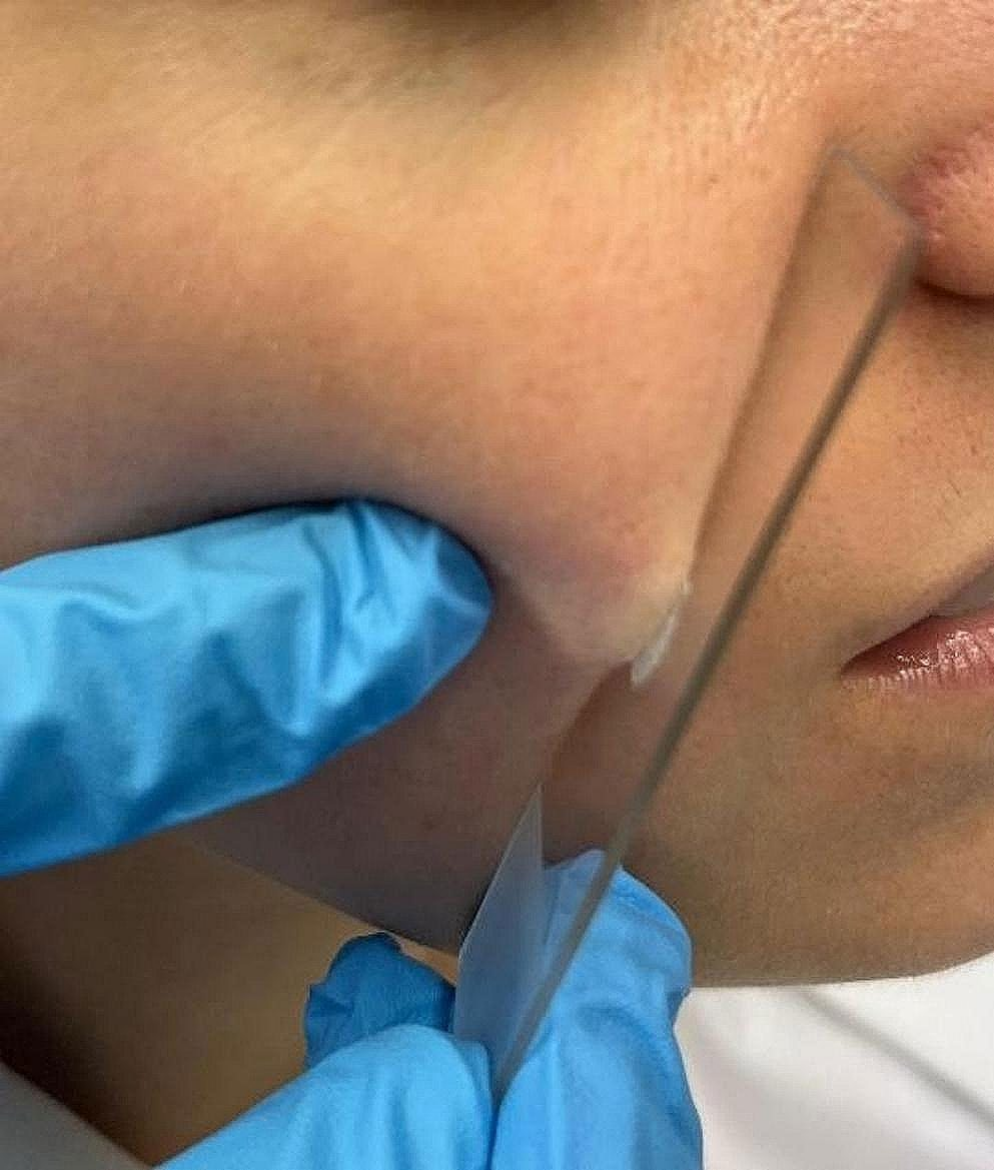
Sampling procedure at the cheek

To study Demodex mites count, SSSB was performed at the cheek and at scalp by a trained dermatologist (Fig. 1). We preferred to take the sample in the occipital area after shaving 1 cm^2^ of skin. SSSB is a sampling method in which 1 cm^2^ of the superficial part of the stratum corneum and of the follicular content of the skin is recovered [4]. Following SSSB test, microscopic examination and PCR were done on the same sample. Microscopic examination was performed with × 10 and × 40 magnifications. Every sample with ≥ 5 Demodex/cm^2^ (D/cm^2^) was considered positive (D+) [4].

**Fig. 2:**
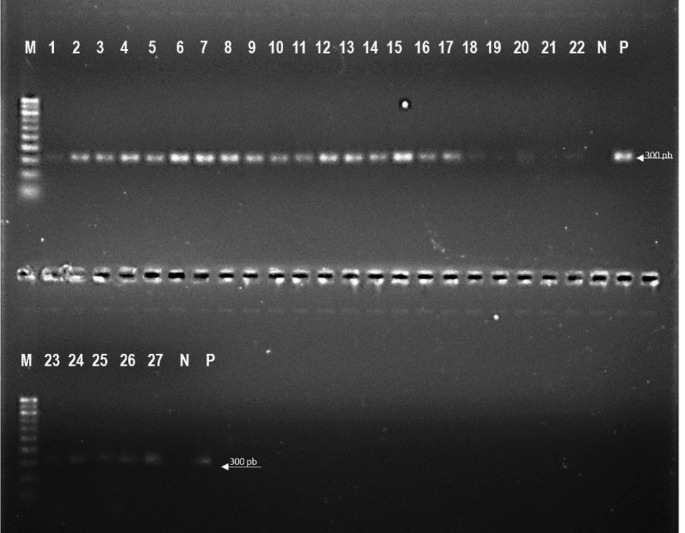
Agarose gel electrophoresis of 16 S rDNA gene products of Demodex mites. Lane M: 100 bp Marker; lane 1–27: Demodex isolates from face and scalp; lane N: Negative control, lane P: Positive control

Molecular identification of Demodex mites from the face and scalp was carried out by amplifying the 18 S ribosomal RNA gene using previously described primers and conditions [9]. DNA was extracted from SSSB using the QIAamp DNA Mini Kit (QIAGEN S.r.l., Italy), following the protocol for the isolation of genomic DNA from tissues. Samples were lysed overnight at 56 °C in ATL buffer and Proteinase K. A PCR mix was prepared for each sample with the following components: 2.5 µl of 10X PCR buffer, 1 µl of 10 mM dNTP mix, 0.8 µl of 50 mM MgCl2, 0.5 µl of each 10 µM primer, 0.2 µl of Invitrogen Platinum Taq DNA Polymerase, and nuclease-free water were combined to make a final volume of 20 µl. The PCR products were visualised using a UV transilluminator (UVITEC Cambridge ‘Gel documentation’ system) after being run on a 1.5% agarose gel stained with SYBR Safe DNA Gel Stain (Invitrogen). The expected PCR product sizes for *D. folliculorum* and *D. brevis* were 382 and 317 bp, respectively (Fig. 2).

## Results

In total, 20 Caucasian patients (17 women, 85% and 3 men, 15%) with an average age of 50.45 years (range 29–72) and 10 controls (8 women, 80%, and 2 men, 30%) with an average age of 49.7 years (range 28–69) were included.

Of the 20 patients, 7 had an almost-clear PPR, 8 had a mild PPR and 5 had a moderate PPR. Six (30%) patients reported itching and 6 (30%) presented dandruff on the scalp. The demographics of cases and controls are summarized in Table 1.

**Table 1:** Cases and controls demographics

Characteristics	Controls (*n* = 10)	Cases (*n* = 20)
Gender		
male	2	3
female	8	17
Age (average)	49.7	50.45
Rosacea severity		
IGA 1	n.a.	7
IGA 2	n.a.	8
IGA 3	n.a.	5

The results of microscopic examination and PCR analysis are summarized in Table 2.

**Table 2:** Comparison positive demodex samples in microscopy and PCR between cases and controls

Techniques	Controls (*n* = 10)	Cases (*n* = 20)	*p*
PCR face			
negative	9	5	*0.001*
positive	1	15	
PCR scalp			
negative	8	7	*0.020*
positive	2	13	
Microscopy face			
negative	9	12	0.09
positive	1	8	
Microscopy scalp			
negative	10	13	*0.033*
positive	0	7	

At the microscopic examination, patients with PPR presented a greater prevalence of Demodex-positive samples than controls at the scalp (35% vs. 0%, *p* = 0.033) and at the face (40% vs. 10%, *p* = 0.09). PCR showed a greater prevalence of Demodex-positive samples at the face (75% vs. 25%, *p* = 0.001) and at the scalp (65% vs. 35%, *p* = 0.020) in patients with PPR.

In total, 16 face samples were positive at microscopic examination or PCR, and 15 scalp samples were positive at microscopic examination or PCR. These cases were considered positive for the presence of Demodex spp.

Microscopy had sensitivity of 50% at the face and of 46.7% at the scalp. PCR had sensitivity of 93.75% at the face and of 86.7% at the scalp.

The positivity of PCR (but not the positivity of microscopic examination) was associated to a higher frequency of papules and pustules at the face (92.3% vs. 42.9%, *p* = 0.031). Patients with positivity of PCR or microscopy at the face had a more frequent positivity at PCR or microscopy at the scalp (93.3% vs. 40%, *p* = 0.032).

The presence of Demodex mites on the scalp (detected by PCR or microscopy) was not associated to the presence of scalp signs and symptoms.

## Discussion

In the literature, there are different studies conducted on patients affected by rosacea to evaluate the frequency of positive Demodex mite sampling on the face using SSSB and microscopic observation [4, 5]. By contrast, the studies conducted to evaluate the presence of Demodex mites on the face and on the scalp with PCR are scanty, and only one study reports the presence of Demodex mites detected with PCR [10].

In our study, we compared the sensitivity of PCR and microscopy in detecting the presence of Demodex mites on SSSB samples collected on the face and scalp.

PCR had a greater sensitivity in both locations and it is a convenient and rapid method. In addition, PCR gives standard results, unlike microscopic observation, which provides much more subjective results.

Moreover, PCR analysis can be applied to a variety of samples, including those that do not require invasive methods, such as skin swab and skin wax. For example, for the diagnosis of scabies, Bae et al. found that PCR is able to detect *Sarcoptes scabiei* mites on skin scrapings with greater sensitivity than microscopy observation [11].

PCR presents several limitations: it is relatively expensive and requires specialized equipment and personnel. Moreover, in our case, PCR was not able to investigate the concentration of mites in the sample, thus not providing information whether Demodex was a saprophyte host or had caused an actual demodicosis linked with its high proliferation at the skin. Nonetheless, real-time PCR could be used in the future, providing quantitative results; further studies are required to compare the results of quantitative PCR and mite count at microscopy.

Traditionally, microscopy constitutes the gold standard for diagnosis of Demodex infestation, because it is a simple, inexpensive, and standardized method [4]. Various samples can be examined by microscopy, such as skin scrapings, etc. [12] and microscopy also allows to observe alive mites and to count them.

Unfortunately, microscopy has several limitations. For example, it is an operator dependent method, which requires specific training and, the sample of choice for microscopic observation, SSSB, can be painful and distressing for the patients, especially when performed on hairy sites, such as the scalp. In addition, microscopy showed to be far less sensitive than PCR in our study, since the detection of mites at optic microscopy is often somewhat difficult, especially in case of limited mite concentration.

The role of Demodex mite proliferation on the scalp is not well known.

Recently, Dall’Oglio et al. have reported Demodex positive scalp skin biopsies in a group of patients with rosacea [13]. Although we found that 9 patients with rosacea presented scalp signs symptoms, we did not find any association between symptoms at the scalp and Demodex mite positivity at microscopy or PCR. This finding may be due to a low number of enrolled patients, however, in our opinion, the scalp could represent a reservoir for the Demodex mites, which can become pathogenetic following the localization on the face. This hypothesis is supported by the greater prevalence of Demodex mites on the scalp in patients with Demodex-positive face samples and by the presence of the same species of Demodex (*Demodex folliculorum*) at the scalp and face.

By adopting a sensitive method, such as PCR, in a large sample, we are able to detect a greater number of Demodex proliferation at the scalp in symptomatic and asymptomatic rosacea patients.

We propose that PCR could be employed in patients with rosacea with or without clear signs of demodicosis of the scalp, as a more sensitive alternative to microscopy, in order to identify the presence of a reservoir of Demodex that could represent a target of treatment. Hopefully, in the future, effective treatments to control Demodex infestation at the scalp may become available, and could be useful to prevent rosacea recurrences soon after the interruption of a standard treatment.

Moreover, PCR could be particularly useful in patients who refuse invasive or painful procedures, since we hypothesize that it could be similarly sensitive on samples other than SSSB, such as skin scrapings and swabs, that can be obtained painlessly. However, the sensitivity of PCR identification of Demodex on such samples should be investigated in further studies.

In conclusion, we demonstrated that PCR is a sensitive method to identify the presence of Demodex on the face and scalp. In particular, we believe that it could be usefully employed on the scalp of recently treated rosacea patients, in order to initiate a prompt antiparasitic treatment of the scalp, that could help prevent future recurrences.

## Data Availability

No datasets were generated or analysed during the current study.
